# A historically controlled, single-arm, multi-centre, prospective trial to evaluate the safety and efficacy of MonoMax^® ^suture material for abdominal wall closure after primary midline laparotomy. ISSAAC-Trial [NCT005725079]

**DOI:** 10.1186/1471-2482-8-12

**Published:** 2008-07-21

**Authors:** Lars Fischer, Petra Baumann, Johannes Hüsing, Christoph Seidlmayer, Markus Albertsmeier, Annette Franck, Steffen Luntz, Christoph M Seiler, Hanns-Peter Knaebel

**Affiliations:** 1 Department of General, Visceral and Transplantation Surgery, University of Heidelberg, Germany; 2 Aesculap AG, Am Aesculap Platz, Tuttlingen, Germany; 3 KKS, University of Heidelberg, Germany; 4 Department of Visceral Surgery, St. Bonifatius-Hospital Lingen, Germany; 5 Department of Surgery, University Munich-Großhadern, Germany; 6 Department of Visceral, Thoracic and Vascular Surgery, Philipps University, Marburg, Germany

## Abstract

**Background:**

Several randomized controlled trials have compared different suture materials and techniques for abdominal wall closure with respect to the incidence of incisional hernias after midline laparotomy and shown that it remains, irrespective of the methods used, considerably high, ranging from 9% to 20%. The development of improved suture materials which would reduce postoperative complications may help to lower its frequency.

**Design:**

This is a historically controlled, single-arm, multi-centre, prospective trial to evaluate the safety of MonoMax^® ^suture material for abdominal wall closure in 150 patients with primary elective midline incisions. INSECT patients who underwent abdominal closure using Monoplus^® ^and PDS^® ^will serve as historical control group. The incidences of wound infections and of burst abdomen are defined as composite primary endpoints. Secondary endpoints are the frequency of incisional hernias within one year after operation and safety. To ensure adequate comparability in surgical performance and recruitment, the 4 largest centres of the INSECT-Trial will participate. After hospital discharge, the investigators will examine the enrolled patients again at 30 days and at 12 ± 1 months after surgery.

**Conclusion:**

This historically controlled, single-arm, multi-centre, prospective ISSAAC trial aims to assess whether the use of an ultra-long-lasting absorbable monofilament suture material is safe and efficient.

**Trial registration:**

NCT005725079

## Background

Midline laparotomy is a standard method for gaining access to the abdominal cavity. However, in spite of a great number of different suture materials and techniques being available to achieve closure of the abdominal wall, the incidence of abdominal wall hernia remains high (ranging from 9% to 20%) [1-3]. The latest data show that 52% of incisional hernias occur within 6 months postoperatively, 68% within one year and 79% within two years after surgery [3,4]. They do not only require most often surgery, but even after adequate treatment, they show recurrence rates up to 45% [5]. Another complication of a complex abdominal wall closure is a burst abdomen occurring in 1% to 3% of the patients within the first days after laparotomy [6-8].

### Rationale

Various randomized controlled trials and four corresponding meta-analyses reported in the literature have compared different suture – slowly absorbable versus rapidly or non-absorbable – materials or different suture – interrupted versus continuous running – techniques for abdominal wall closure [9-14]. These studies suggest that, using a running suture technique, slowly absorbable monofilament sutures may be the best choice for abdominal wall closure. As one possible reason for the still relevant incidence of incisional hernias, experimental research has demonstrated that, one year after a midline laparotomy, the abdominal fascia retains only 70% of its original strength, suggesting that adequate abdominal wall closure preferably needs a longer-lasting supporting suture [4]. Even though synthetic absorbable sutures such as polyglactin, polyglycolic and polydioxanone are slowly absorbable, they are fully absorbed after 70 to 180 days. Therefore, an ultra-long-term absorbable suture material may help to reduce the above mentioned postoperative complications such as incisional hernia or burst abdomen.

### Purpose

The ISSAAC-Trial, a prospective multi-centre historically controlled trial, was designed to evaluate the clinical safety and efficacy of MonoMax^®^, a new suture material. MonoMax^® ^is an ultra-long-lasting, absorbable, flexible and elastic monofilament with superior initial strength and predictable constant degradation rate. Compared to other absorbable monofilament suture materials, MonoMax^® ^shows superiority in knot and long-lasting linear tensile strength retention and provides additional security, indicating that MonoMax^® ^might be very well suitable for abdominal wall closure. To ensure optimal comparability with the study population of the INSECT-Trial as well as fast patient recruitment, only the four centres most successful in recruiting patients for the INSECT-Trial will enrol patients for the ISSAAC-Trial.

## Methods/Design

### Study objectives

The aim of the ISSAAC-Trial is to show the safety and efficacy of MonoMax^® ^suture material. To assess its safety, parameters such as wound infections and re-operation due to burst abdomen will be evaluated. It is expected that the frequency of both parameters may be equal or lower in the ISSAAC patient cohort than in the INSECT patient cohort. To assess its efficacy, the frequency of abdominal hernias at 12 ± 1 months after surgery is taken as parameter and is also expected to may be equal or lower than in the INSECT-Trial [15,16].

The hypothesis would be that that the frequency of wound infection and of re-operation due to burst abdomen until day of discharge are equal or lower in the patient cohort enrolled in the ISSAAC-Trial compared to patients included in the INSECT-Trial.

For the ISSAAC-Trial, a standardized surgical technique of abdominal wall closure with continuous monofilament sutures is being used as it was in the INSECT-Trial. Thus, the results obtained from both trials will be comparable. To this end, only patients operated with the use of either MonoPlus^® ^or PDS^® ^as suture material during the INSECT-Trial will be included in the control group, excluding the patients randomized to interrupted sutures.

The composite primary endpoints of the ISSAAC-Trial are the frequency of wound infection, defined as redness, wound dehiscence with secretion (putrid or caliginous, smelly fluid) and/or microbiologic evidence of bacterial contamination, and the frequency of re-operation due to burst abdomen until day of discharge (see Table 1).

**Table 1:** Definition of complications

Complication	Definition
Burst abdomen	Postoperatively missing continuity of the abdominal fascia in combination with a wound dehiscence and consecutive relapse operation.
Wound infection	Redness, wound dehiscence with secretion either of putrid or caliginous, smelly fluid and/or mircobiologic evidence of bacterial contamination.
Incisional hernia	A hernia is present if the ultrasound shows a fascial gap and a protruding hernial sac and the clinical exam is consistent with a hernia.
Complicated wound healing	Incision has not completely closed. Necrosis of the wound edge or primary or secondary dehiscence occurs. Formation of seroma, fistula, necrosis, and bleeding.

The secondary endpoints of the ISSAAC-Trial are the frequency of abdominal hernias at 12 ± 1 months after surgery, the frequency of wound-infection and complicated wound healing at 30 days after surgery, and the length of the hospital stay.

The incidence of incisional hernia within 12 ± 1 months after the operation diagnosed by physical and ultrasound examination is considered as secondary endpoint. A hernia is deemed present if the ultrasound examination shows a fascial gap and a protruding sac and if confirmed by the clinical examination. Assessment and documentation will be performed by a person who is familiar with the examination of the abdominal wall, but not involved in the surgical procedure. The ultrasound examination will be carried out by an investigator who has at least 6 months training in this method.

### Ethics and informed consent

The final protocol was approved by the Ethics Committee of the University of Heidelberg Medical School. Secondary approval was obtained from all local ethics committees responsible for the participating centres. All patients eligible will be asked whether they are willing to participate in the trial and informed about the purpose of the trial, the operative procedures as well as their options and risks. Written informed consent will be obtained from all patients participating in the trial.

### Intervention

#### Surgical technique

The operation begins with a skin incision performed with the electric cautery which penetrates through the subcutaneous layer. Next, the abdominal fascia is separated in the midline line with the electric cautery. Then, the incision of the peritoneum is made with the scissors and completed with the electric cautery. The surgical procedure is carried out in the usual fashion and according to local standards regarding the indication for the intervention.

Starting either from the cranial end or from the caudal end of the wound, the abdominal wall is closed by placing four Mikulicz or equivalent clamps at the edges of the abdominal fascia and a continuous all-layer suture using two MonoMax^® ^loops (USP1/Aesculap AG, Tuttlingen, Germany). Before use, in order to avoid breakage of the material, the monofilament suture MonoMax^® ^has to be stretched once by the assisting nurse/operation technician. The first stitch should be anchored cranially and caudally of the incision. The distance to the edge of the fascia and the distance between two stitches should not exceed 2 cm to 2.5 cm. After having closed half of the wound, the surgeon cuts one end of the loop immediately below the needle, passes it then from the opposite edge of the fascia and ties both ends with at least four knots. He proceeds in the same manner with the loop from the caudal end of the wound which intersects the other loop at the middle of the incision, both sutures lines overlapping at least 2 cm. For every patient two loops must be used, irrespective of the length of the wound. After completion of the fascia closure, the loop is cut directly underneath the needle.

No subcutaneous or drainage is inserted. The skin is closed with skin clips or interrupted sutures with monofilament non-absorbable sutures. At the end of the intervention, the length of the incision is measured in cm.

During the first investigator meeting (November 22^th^, 2007, Stuttgart, Germany), all participating trial centres were retrained in the required technique using abdominal wall models of mini-pigs and provided with a CD-ROM demonstrating the required surgical technique. Furthermore, they have continuous access to technical support by suture product specialists from Aesculap AG for all suture-related issues and questions.

#### Postoperative intervention

After discharge from the hospital, the investigators will examine the patients at 30 days and at 12 ± 1 months after surgery. All complications leading to re-operation due to burst abdomen, wound infection and complicated wound healing will be documented within 30 days after surgery. At one year postoperatively, the incidence of incisional hernias will be recorded by physical and abdominal ultrasound examination. In total, the trial will last 12 ± 1 months for each patient (see Table 2).

**Table 2:** Flow Chart ISSAAC Trial

	Visit 1 (Screening)	Visit 2 (day 0)	Visit 3 (day 2 ± 1)	Visit 4 (day of discharge or the day before)	Visit 5 (day 30 ± 5)	Visit 6 (12 ± 1 months post surgery)
Informed consent	X					
Demographic data*	X					
BMI	X					
Smoker/non-smoker	X					
Inclusion/exclusion	X					
Medical history	X					
Physical examination	X		X1)	X1)	X1)	X2)
Reason for surgery	X					
Surgery		X				
Board certified surgeon		X				
Deviations from surgical procedure as described in protocol		X				
Ultrasound of the abdominal wall						X
Abdominal Adverse Event/Serious Adverse Event		X	X	X	X	X
Re-operation due to burst abdomen necessary?			X	X	X	X
Wound infection			X	X	X	
discharge				X		

1) of the abdomen and the laparotomy wound

2) of the abdomen

* date of birth, gender, weight, height

### Clinical sites

The selected sites were most successful in enrolling patients for the INSECT-Trial. Therefore four centres located in Germany will recruit 150 patients (for all inclusion and exclusion criteria see Table 3). All investigators are hospital-based general surgeons.

**Table 3:** Eligibility Criteria

Inclusion criteria	Exclusion criteria
Age equal or greater than 18 years	Peritonitis
Expected survival time more than 12 months	Emergency surgery
Patients undergoing primary and elective midline laparotomy (patients with prior laparoscopy or abdominal operation via paramedian incision (e.g. appendectomy) may be included in the trial	Coagulopathy (= A group of disorders of the blood clotting (coagulation) system in which bleeding is prolonged and excessive with abnormal values in the blood laboratory • Lack of compliance
BMI < 35	Severe psychiatric or neurologic disease
Expected length of skin incision > 15 cm	Lack of informed consent
	Participation in another intervention with interference of intervention and outcome of this trial
	Drug- and/or alcohol-abuse according to local standard
	Current immunosuppressive therapy (more than 40 mg of a corticoid per days or azathioprin
	Chemotherapy within two 2 before operation
	Radiotherapy of the abdomen completed less than 8 weeks before operation
	Inability to understand and to follow the instructions given by the investigator (e.g. insufficient command of language, dementia, lack of time)
	Pregnant or breast-feeding woman (according to information given by the patient)
	Patients who have been committed to an institution by virtue of an order issued either by the courts or by an authority

### Safety aspects

The term "adverse event" covers any sign, symptom, syndrome, illness that appear or worsen in a subject during the period of observation in the clinical trial and that may impair the well-being of the subject. The term also covers laboratory findings of other diagnostic procedures that are considered to be clinically relevant.

A "serious adverse event" is any adverse event occurring at any time during the period of observation that results in death, is immediately life-threatening, requires or prolongs hospitalization, results in persistent or significant disability or incapacity.

All adverse events occurring in the abdominal region between the beginning of wound closure and the last visit of the patient at 12 months postoperatively must be documented on the appropriate AE form in the CRF, excluding adverse events that affect other body systems as they are not related to the intervention. Complications such as burst abdomen, wound infection, complicated wound healing, presence of incisional hernia are defined as adverse events (for definition see Table 1). Re-operation due to burst abdomen is classified as a serious adverse event.

Analysis of safety related data performed with respect to:

- Frequency of SAEs

- Frequency of SAEs stratified by severity

- Frequency of SAEs stratified by causality.

The sponsor will notify the competent authorities, ethics committees and all investigators concerned of unexpected SAEs and unexpected adverse device effects in line with applicable regulatory requirements.

As the adverse event definition used in the INSECT-Trial was broader, two surgeons will independently select from this trial's list of adverse events the adverse events to be taken for comparison in the present trial in accordance with the established definition.

### Statistical considerations and sample size estimation

Patients are included for primary analysis if information about complications is available up to the day of discharge. Non-compliant and compliant patients will be compared with respect to age and sex distribution.

About 80 patients from the INSECT-Trial will serve as control group. Using the 1:4 rule, up to 320 patients could be collected in a sensible manner. Within a reasonable time-frame of 1 year, 150 patients could be enrolled into the ISSAAC-Trial. Assuming a drop-out rate of 7%, 140 patients could be subjected to an analysis with respect to the primary endpoints. This would be sufficient to construct a 90% confidence interval around the estimate that would not cover the value of 25% with a power of 10% when the true event rate is at 15%.

It is expected that the historical control group will comprise about 130 patients who had been enrolled into the INSECT-Trial. To make the comparison with the historical control group as meaningful as possible (using an "information per patient" metric), the size of the MonoMax^® ^group should not exceed the size of the control group by too much und has been accordingly established at 150 patients, allowing for 10 drop-outs.

Each participating centre should add at least 1/4 to the patients it had recruited during the INSECT-Trial, e.g. Marburg at least 7 patients and the other three at least 10 patients each. In order to avoid a centre effect, no participating hospital should include more than 80 patients.

The level of significance for the confidence intervals is set at 95 and 90% in order to perform a one-sided test of significance against the 25% event rate stated in the null hypothesis.

### Trial organization, coordination and registration

The ISSAAC-Trial is initiated and sponsored by BBraun Aesculap and conducted by Aesculap AG in cooperation with the Coordinating Center for Clinical Trials (KKS) in Heidelberg. The KKS Heidelberg is responsible for monitoring, biometrics, database, and project management. The Sponsor's role is limited to supplying the participating trial centre with the suture material and surgical support. It is not involved in the data base management. It has taken out an insurance policy to cover all patients taking part in the trial.

The trial is coordinated by the Coordinating Center for Clinical Trials in cooperation with BBraun Aesculap. Both were responsible for management and registration (Identifier Number NCT 0005725079, ) and all trial related meetings.

The trial is performed according to the Declaration of Helsinki in its current German version, the national medical device law and the guidelines for Good Clinical Practice (GCP), as applicable.

### On-site monitoring

Authorized, qualified representatives of the Sponsor will visit the investigational sites at regular intervals to verify adherence to protocol and local legal requirements, perform source data verification and assist the Investigator in his trial related activities.

During recruitment of patients, the participating study centres will be monitored according to Good Clinical Practice (GCP) guidelines. A monitor from the Coordinating Center for Clinical Trials will audit the data.

### Data management and quality assurance

Data is entered in prepared electronic CRFs which form a front end to a database. The Clinical Database Management System MACRO (Infermed Ltd., London, UK) is used for planning and entering data into the database. Range and plausibility checks are programmed into the database. An audit trail ensures that original entries remain accessible after correction. Data entry is be performed by personnel trained on the use of the system during the monitor's initiation visit.

Data reported on the CRF derived from source documents should be consistent with the source documents or the discrepancies should be explained. Within two weeks after completion for each patient, the Investigator should confirm that he has completed, signed the CRFs and made them available for full inspection by the clinical monitor.

Enrolment must be reported immediately by the investigators by fax on prepared forms directly to the KKS.

### Closing of the clinical data and follow-up database

The clinical database, including all information until 12 months postoperatively, will be closed after the 12 months follow-up visit of the last patient enrolled into the ISSAAC-Trial. There will be an interim analysis after the 30 days follow-up visit of the last enrolled patient which includes the combined primary endpoint and the secondary endpoints, i.e. wound infections and wound healing disorders after 30 days of surgery. The results of the other secondary endpoint, incisional hernia at 12 months postoperatively, will be evaluated accordingly.

### Current status and planning

The study protocol for this trial was completed in August 2007. A first investigator meeting was held on 22^th ^November, 2007 in Stuttgart, Germany. Preparation of all study related-material was brought to an end in November 2007. In December 2007, following completion of contracts and approval of local ethics committees, all centres were initiated, the first patient being recruited on the 5^th ^December 2007. Currently all 4 centres are recruiting patients with a goal of 150 patients. Assuming an overall enrolment of 25 patients per month, the end of recruitment is expected for June 2008.

## Discussion

An "ideal" suture material would help to prevent the occurrence of incisional hernias and other complications such as wound infections, wound pain and suture sinus to a minimum. Although various randomized controlled clinical trials have been reported in the literature addressing the use of different suture materials and techniques for abdominal wall closure after midline laparotomy, no ideal combination of suture material or technique has been found so far [9,12-14,17].

Several of these trials compared slowly-absorbable versus fast-absorbable or non-absorbable suture materials and others interrupted versus continuous running suture techniques [9,12-14,16-18]. They reported a significant higher incidence of wound pain and suture sinus formation where non-absorbable suture material was used. The results also showed that closing the abdominal wall closure with continuous rapidly absorbable suture material was correlated with a significantly higher occurrence of incisional hernias compared to closure by continuous slowly-absorbable or non-absorbable suture material [12,22]. However, no statistically significant differences in outcome features of wound dehiscence and wound infection were found when various suture materials were compared [13,14]. It has been suggested a correlation between postoperative wound infection and development of incisional hernias [17]. Thus, the prevention of wound infection may be more important in avoiding incisional hernias than the kind of suture material or the type of closure technique [19-21].

Most authors suggest that a slowly absorbable monofilament suture material is superior to a non-absorbable suture material for closure of the abdominal wall. Furthermore, the presumably ideal suture technique appears to be a mass closure using a continuous running suture technique with an adequate suture length to wound length ratio of at least 4:1 [14,22,23]. However, there is no standard technique generally accepted as best or rather safest for closing the abdominal wall after primary midline laparotomy. This was one of the reasons to design and conduct the INSECT-Trial with the aim to answer the question of the best method to perform this intervention [16]. Using three different closure methods, the frequency of incisional hernias within one year postoperatively was compared as primary endpoint.

Synthetic absorbable sutures which have become available over the last decade have the advantage that they are degraded by the body system and fully absorbed within 70 to 180 days. However, they lose 50% of their tensile strength already after 14 to 30 days [24]. Therefore, they may be not the optimal suture material for abdominal wall closure. The ISSAAC-Trial was designed to investigate whether the use of an ultra-long-lasting absorbable monofilament suture material is safe and prevents the occurrence of incisional hernias after primary midline laparotomy.

Several potential risk factors may have an influence on the occurrence of incisional hernias such as complicated wound healing, wound infections, obesity, chronic bronchitis or diabetes mellitus [4,25-28]. However, wound infection remains the most significant early postoperative complication in 3 to 21% of patients undergoing midline laparotomy [4,19,21,29,30]. Thus, the prevention of wound healing complications should reduce substantially the incidence of incisional hernias. Studies comparing slowly absorbable versus rapidly absorbable suture material for closing abdominal wounds showed that none of the suture materials provided satisfactory results in preventing wound healing complications. In order to evaluate the safety of MonoMax^®^, we chose as primary combined endpoint for our trial the frequency of wound infections, the frequency of complicated wound healing and the frequency of re-operation due to burst abdomen until day of discharge and defined as secondary endpoints the frequency of wound infection and healing disorders at 30 days after surgery. Since the literature shows that 50 to 70% of incisional hernias develop within one year postoperatively, we chose an additional secondary endpoint, that is the frequency of incisional hernia one year after surgery, as efficacy variable [4].

In the ISSAAC-Trial, a highly standardized surgical procedure will be used along with the suturing technique already used in the INSECT-Trial. Thus, it is reasonable to compare the results from the present trial with the data already collected during the earlier study. Because ISSAAC uses continuous monofilament suturing material, only patients enrolled in the INSECT-Trial who underwent the same treatment with MonoPlus^® ^and PDS^® ^will serve as control group.

## Conclusion

The ISSAAC trial is a historically controlled, single arm, multi-centre, prospective surgical trial to evaluate the safety and efficacy of a long-lasting suture material, MonoMax^®^, for abdominal wall closure after primary midline laparotomy. As clinical relevant conclusion, the ISSAAC-Trial aims to find out whether the use of MonoMax^® ^will reduce the rate of wound infections and subsequently lower the incidence of incisional hernias.

## Abbreviations

AE: Adverse event; AWC: Abdominal wall closure; CRF: Case Report Form; GCP: Good Clinical Practice; INSECT: Interrupted or continuous slowly absorbable sutures – evaluation of abdominal closure techniques; KKS: Coordinating Center for Clinical Trials; PDS: Polydioxanone; SAE: Serious Adverse Event.

## Competing interests

This study and its publication are sponsored by Aesculap AG, Germany.

## Authors' contributions

PB and HPK (BBraun Aesculap, Tuttlingen, Germany) designed, managed and conducted the trial together with the KKS Heidelberg (SL) and LF. LF wrote the manuscript together with PB and HPK. JH is involved as biostatistician. The other authors are participating actively in the recruitment of the patients. All authors have read and approved this manuscript.

## Pre-publication history

The pre-publication history for this paper can be accessed here:



## References

[B1] Conze J, Klinge U, Schumpelick V (2005). Incisional hernia. Chirurg.

[B2] Sugerman HJ, Kellum JM, Reines HD, DeMaria EJ, Newsome HH, Lowry JW (1996). Greater risk of incisional hernia with morbidly obese than steroid-dependent patients and low recurrence with prefascial polypropylene mesh. Am J Surg.

[B3] Mudge M, Hughes LE (1985). Incisional hernia: a 10 year prospective study of incidence and attitudes. Br J Surg.

[B4] Hoer J, Lawong G, Klinge U, Schumpelick V (2002). Factors influencing the development of incisional hernia. A retrospective study of 2,983 laparotomy patients over a period of 10 years. Chirurg.

[B5] Flum DR, Horvath K, Koepsell T (2003). Have outcomes of incisional hernia repair improved with time? A population-based analysis. Ann Surg.

[B6] Luijendijk RW, Lemmen MH, Hop WC, Wereldsma JC (1997). Incisional hernia recurrence following "vest-over-pants" or vertical Mayo repair of primary hernias of the midline. World J Surg.

[B7] Paul A, Lefering R, Kohler L, Eypasch E (1997). [Current practice of incisional hernia reconstruction in Germany]. Zentralbl Chir.

[B8] Manninen MJ, Lavonius M, Perhoniemi VJ (1991). Results of incisional hernia repair. A retrospective study of 172 unselected hernioplasties. Eur J Surg.

[B9] van 't RM, Steyerberg EW, Nellensteyn J, Bonjer HJ, Jeekel J (2002). Meta-analysis of techniques for closure of midline abdominal incisions. Br J Surg.

[B10] Krukowski ZH, Cusick EL, Engeset J, Matheson NA (1987). Polydioxanone or polypropylene for closure of midline abdominal incisions: a prospective comparative clinical trial. Br J Surg.

[B11] Cameron AE, Parker CJ, Field ES, Gray RC, Wyatt AP (1987). A randomised comparison of polydioxanone (PDS) and polypropylene (Prolene) for abdominal wound closure. Ann R Coll Surg Engl.

[B12] Weiland DE, Bay RC, Del SS (1998). Choosing the best abdominal closure by meta-analysis. Am J Surg.

[B13] Hodgson NC, Malthaner RA, Ostbye T (2000). The search for an ideal method of abdominal fascial closure: a meta-analysis. Ann Surg.

[B14] Rucinski J, Margolis M, Panagopoulos G, Wise L (2001). Closure of the abdominal midline fascia: meta-analysis delineates the optimal technique. Am Surg.

[B15] Knaebel HP, Kirschner MH, Reidel MA, Buchler MW, Seiler CM (2006). Operative standardization in randomized controlled surgical trials. Meeting of the INSECT trial. Chirurg.

[B16] Knaebel HP, Koch M, Sauerland S, Diener MK, Buchler MW, Seiler CM (2005). Interrupted or continuous slowly absorbable sutures - design of a multi-centre randomised trial to evaluate abdominal closure techniques INSECT-trial [ISRCTN24023541]. BMC Surg.

[B17] Wissing J, van Vroonhoven TJ, Schattenkerk ME, Veen HF, Ponsen RJ, Jeekel J (1987). Fascia closure after midline laparotomy: results of a randomized trial. Br J Surg.

[B18] Hsiao WC, Young KC, Wang ST, Lin PW (2000). Incisional hernia after laparotomy: prospective randomized comparison between early-absorbable and late-absorbable suture materials. World J Surg.

[B19] Israelsson LA, Jonsson T, Knutsson A (1996). Suture technique and wound healing in midline laparotomy incisions. Eur J Surg.

[B20] Yahchouchy-Chouillard E, Aura T, Picone O, Etienne JC, Fingerhut A (2003). Incisional hernias. I. Related risk factors. Dig Surg.

[B21] Irvin TT, Stoddard CJ, Greaney MG, Duthie HL (1977). Abdominal wound healing: a prospective clinical study. Br Med J.

[B22] Israelsson LA, Jonsson T (1993). Suture length to wound length ratio and healing of midline laparotomy incisions. Br J Surg.

[B23] O'Dwyer PJ, Courtney CA (2003). Factors involved in abdominal wall closure and subsequent incisional hernia. Surgeon.

[B24] Miles JS (1986). Use of polydioxanone absorbable monofilament sutures in orthopedic surgery. Orthopedics.

[B25] Cassar K, Munro A (2002). Surgical treatment of incisional hernia. Br J Surg.

[B26] Sorensen LT, Hemmingsen UB, Kirkeby LT, Kallehave F, Jorgensen LN (2005). Smoking is a risk factor for incisional hernia. Arch Surg.

[B27] Klinge U, Conze J, Limberg W, Brucker C, Ottinger AP, Schumpelick V (1996). Pathophysiology of the abdominal wall. Chirurg.

[B28] Junge K, Rosch R, Klinge U, Schwab R, Peiper C, Binnebosel M, Schenten F, Schumpelick V (2006). Risk factors related to recurrence in inguinal hernia repair: a retrospective analysis. Hernia.

[B29] Gys T, Hubens A (1989). A prospective comparative clinical study between monofilament absorbable and non-absorbable sutures for abdominal wall closure. Acta Chir Belg.

[B30] Osther PJ, Gjode P, Mortensen BB, Mortensen PB, Bartholin J, Gottrup F (1995). Randomized comparison of polyglycolic acid and polyglyconate sutures for abdominal fascial closure after laparotomy in patients with suspected impaired wound healing. Br J Surg.

